# Supplementary material for: Antimicrobial Activity and Chemical Composition of Essential Oils against Pathogenic Microorganisms of Freshwater Fish
Source: Plants (Basel). 2021 Jun 22;10(7):1265. doi: 10.3390/plants10071265 (PMC8309039; doi:10.3390/plants10071265)
Supplement: Supplementary file 1 [file plants-10-01265-s001.zip › plants-1227051-supplementary.pdf]

Supplemental file 1. Chemical composition of essential oils (%) *.

Essential Oil	Components[a]	Percentage of Components[b]
***Amyris balsamifera* L.**	α-gurjunene	2.4
	(*E*)-caryophyllene	1.3
	α-curcumene	3.2
	α-zingiberene	1.9
	β-dihydro agarofuran	1.1
	β-bisabolene	1.9
	β-sesquiphellandrene	2.7
	selina-3,7(11)-diene	1.5
	elemol	11.5
	τ-cadinol	33.4
	β-eudesmol	1.9
	α-eudesmol	9.6
	α-cadinol	1.3
	7-epi-α-eudesmol	7.7
	selin-11-en-4-α-ol	11.3
	drimenol	2.6
***Boswellia carterii* L.**	α-thujene	4.2
	α-pinene	37.0
	camphene	1.3
	sabinene	4.1
	β-pinene	1.9
	β-myrcene	2.6
	p-cimene	6.3
	α-limonene	19.8
	trans-pinocarveol	1.0
	bornyl acetate	1.0
	β-elemene	2.2
	(*E*)-caryophyllene	2.5
	α-amorphene	1.1
	β-selinene	1.5
	caryophyllene oxide	1.6
***Canarium luzonicum* Miq.(H)**	sabinene	5.1
	α-phellandrene	12.6
	p-cimene	4.9
	α-limonene	39.7
	α-terpinolene	1.2
	4-terpinenol	1.2
	α-terpineol	3.7
	elemol	20.8
	γ-eudesmol	1.3
	α-eudesmol	1.5

Supplemental file 1. Cont.

**Essential Oil**	**Components**[a]	**Percentage of Components**[b]
***Amyris balsamifera* L.**	α-gurjunene	2.4
	(*E*)-caryophyllene	1.3
	α-curcumene	3.2
	α-zingiberene	1.9
	β-dihydro agarofuran	1.1
	β-bisabolene	1.9
	β-sesquiphellandrene	2.7
	selina-3,7(11)-diene	1.5
	elemol	11.5
	τ-cadinol	33.4
	β-eudesmol	1.9
	α-eudesmol	9.6
	α-cadinol	1.3
	7-epi-α-eudesmol	7.7
	selin-11-en-4-α-ol	11.3
	drimenol	2.6
***Boswellia carterii* L.**	α-thujene	4.2
	α-pinene	37.0
	camphene	1.3
	sabinene	4.1
	β-pinene	1.9
	β-myrcene	2.6
	p-cimene	6.3
	α-limonene	19.8
	trans-pinocarveol	1.0
	bornyl acetate	1.0
	β-elemene	2.2
	(*E*)-caryophyllene	2.5
	α-amorphene	1.1
	β-selinene	1.5
	caryophyllene oxide	1.6
***Canarium luzonicum* Miq.(H)**	sabinene	5.1
	α-phellandrene	12.6
	p-cimene	4.9
	α-limonene	39.7
	α-terpinolene	1.2
	4-terpinenol	1.2
	α-terpineol	3.7
	elemol	20.8
	γ-eudesmol	1.3
	α-eudesmol	1.5

Table 1. *Cont.*

Essential Oil	Components[a]	Percentage of Components[b]
**_Cinnamomum camphora_ Nees & Eberm**	α-thujene	2.3
	α-pinene	12.2
	camphene	1.4
	sabinene	5.6
	β-pinene	2.9
	β-myrcene	1.2
	o-cimene	12.1
	α-limonene	25.1
	1,8-cineole	35.2
**_Cinnamomum camphora_ var. _linalolifera_**	linalool	98.1
**_Citrus aurantium_ L.**	α-pinene	1.1
	β-myrcene	1.0
	α-limonene	1.2
	1,8-cineole	5.0
	(*E*)-β-ocimene	1.3
	linalool	22.8
	α-terpineol	6.6
	nerol	1.2
	linalool acetate	48.5
	neryl acetate	2.7
	geranyl acetate	4.6
**_Gaultheria procumbens_ L.**	methyl salicylate	97.6
**_Litsea cubeba_ Pers.**	α-pinene	1.7
	sabinene	1.9
	β-pinene	2.3
	6-methyl-5-hepten-2-one	1.3
	α-limonene	14.3
	1,8-cineole	1.9
	α-terpinolene	1.6
	neral	29.5
	geranial	39.4
**_Melaleuca leucadendron_ L.**	α-thujene	5.6
	α-pinene	5.4
	β-pinene	2.1
	p-cimene	3.3
	α-limonene	6.9
	1,8-cineole	49.0
	γ-terpinene	2.4
	α-terpinolene	1.0
	α-terpineol	7.8
	α-terpinyl acetate	1.3
	(*E*)-caryophyllene	4.3
	β-selinene	1.8
	α-selinene	1.2

Table 1. *Cont.*

Essential Oil	Components[a]	Percentage of Components[b]
***Melaleuca ericifolia* Smith.**	α-pinene	1.0
	o-cymene	1.3
	α-limonene	1.3
	1,8-cineole	16.9
	g-terpinene	1.5
	cis-linalool oxide	1.2
	trans-linalool oxide	1.4
	linalool	47.5
	camphor	1.3
	4-terpinenol	2.9
	α-terpineol	5.0
	aromadendrene	5.3
	ledene	1.5
	caryophyllene oxide	1.2
***Pogostemon cabli* L.**	β-patchoulene	3.0
	β-elemene	2.2
	(*E*)-caryophyllene	3.4
	α-guaiene	14.3
	seychellene	6.9
	α-patchoulene	6.8
	β-selinene	2.2
	α-selinene	3.3
	α-bulnesene	21.3
	caryophyllene oxide	1.1
	5-epi-7-epi-α-eudesmol	1.1
	patchouli alcohol	31.0
***Citrus limon* (L.)**	α-pinene	2.6
	sabinene	2.8
	β-pinene	13.3
	β-myrcene	2.0
	p-cimene	1.0
	α-limonene	58.9
	γ-terpinene	11.2
	neral	1.1
	geranial	1.8
***Santalum album* L.**	α-santalene	5.03
	α-bergamotene	9.68
	α-santalal	2.54
	α-santalol	59.0
	β-santalol	9.02
	Lanceol	1.93
	(E)-nuciferol	1.74
	7-(5-hexynyl)-tricyclo[4.2.2.0(2,5)]dec-7-ene	1.38

Table 1. *Cont.*

Essential Oil	Components[a]	Percentage of Components[b]
***Vetiveria zizanoides* (L.)**	3,3,5,6,8,8-Hexamethyltricyclo[5.1.0.0(2,4)]oct-5-ene	1.02
	Tricyclo[6.3.0.0(1,5)]undec-2-en-4-one, 5,9-dimethyl	2.00
	1,2,4,5-tetraethylbenzene	4.39
	α-muurolene	1.89
	α-cadinene	1.74
	Selina-5,11-diene	1.50
	δ-cadinene	1.57
	α-vetispirene	1.77
	β-vetispirene	2.03
	γ-vetivenene	3.30
	β-vetivenene	7.42
	Valencen	2.19
	3,5,11-Eudesmatriene	1.65
	7,7-dichlorobicyclo[3.2.0]hept-2-en-6-one	1.22
	γ-himachalene	2.63
	Ziza-6(13)-en-12-al	1.02
	Khusiol	1.12
	β-guaiene	4.43
	Cyclocopacamphenol	1.66
	Zizanol	1.03
	(E)-isovalencenal	1.37
	Vetiselinenol	1.41
	Vetiverol	2.22
	Khusenol	5.24
	Vetiverone	3.02
	β-costol	3.52
	Khusenic acid	4.17

Note: * listed are the components that represented min. 1%, [a] Identified compounds, [b] compounds identified in amounts.
